# Combination Therapy of Chemoembolization and Hepatic Arterial Infusion Chemotherapy in Hepatocellular Carcinoma with Portal Vein Tumor Thrombosis Compared with Chemoembolization Alone: A Propensity Score-Matched Analysis

**DOI:** 10.1155/2021/6670367

**Published:** 2021-07-14

**Authors:** Bao-Jiang Liu, Song Gao, Xu Zhu, Jian-Hai Guo, Fu-Xin Kou, Shao-Xing Liu, Xin Zhang, Xiao-Dong Wang, Guang Cao, Hui Chen, Peng Liu, Lin-Zhong Zhu, Hai-Feng Xu, Ren-Jie Yang

**Affiliations:** Department of Interventional Therapy, Peking University Cancer Hospital and Institute, Key Laboratory of Carcinogenesis and Translational Research (Ministry of Education), 52 Fucheng Road, Haidian District, Beijing 100142, China

## Abstract

**Background:**

Survival of patients with portal vein tumor thrombosis (PVTT) is extremely poor; transarterial chemoembolization (TACE) is a treatment for patients with HCC and PVTT. Some studies showed that hepatic arterial infusion chemotherapy (HAIC) might improve the survival of HCC with PVTT. There were few researches of combining TACE with HAIC for patients with HCC and PVTT.

**Aim:**

This study was aimed at comparing overall survival (OS) and progression-free survival (PFS) following treatment with conventional transarterial chemoembolization plus hepatic arterial infusion chemotherapy (cTACE-HAIC) or conventional transarterial chemoembolization (cTACE) alone in patients with hepatocellular carcinoma (HCC) and portal vein tumor thrombosis (PVTT).

**Methods:**

From January 2011 to December 2016, 155 patients with HCC and PVTT who received cTACE-HAIC (cTACE-HAIC group) (*n* = 86) or cTACE alone (cTACE group) (*n* = 69) were retrospectively evaluated. Propensity score matching (PSM) reduced the confounding bias and yielded 60 matched patient pairs. The tumors' responses were evaluated using the modified response evaluation criteria in solid tumors (mRECIST). OS and PFS of groups were compared using the Kaplan-Meier method, log-rank test, and Cox proportional hazard regression models.

**Results:**

The median follow-up duration was 93 months (range: 1–93 months). The cTACE-HAIC group's OS (9.0 months) and PFS (6.0 months) were significantly longer than the cTACE group's OS (5.0 months) and PFS (2.0 months) (*p* = 0.018 and *p* = 0.045, respectively) in the matched cohort. Multivariate analyses showed that cTACE-HAIC was independently associated with OS (hazard ratio (HR) 0.602, *p* = 0.010) and PFS (HR 0.66, *p* = 0.038). The matched groups did not differ regarding grade 3 or 4 adverse events.

**Conclusion:**

cTACE-HAIC was superior to cTACE alone regarding OS and PFS in patients with HCC and PVTT. Treatment-associated toxicities were generally well tolerated.

## 1. Introduction

Hepatocellular carcinoma (HCC) is a common malignancy and the fourth leading cause of cancer-related death globally [1]. The presence of portal vein tumor thrombosis (PVTT) is an important prognostic factor among patients with HCC [2], and 10–40% of patients have macroscopic PVTT on diagnosis. Survival rates among patients with PVTT are extremely poor, and the median overall survival (OS) for those with untreated tumors ranges from 1 to 2 months [3, 4].

Transarterial chemoembolization (TACE) is a treatment for patients with HCC and PVTT [5, 6]. In addition, TACE can prolong the survival of patients with HCC and PVTT [7, 8], and it is often used as the first-line treatment in eastern countries [9–11]; however, the treatment response rates for TACE are low, with a median OS of 3.0–7.5 months [8, 12, 13].

A randomized, prospective, comparative study of patients with advanced HCC and PVTT [14] showed that hepatic arterial infusion chemotherapy (HAIC) might improve the median OS and PFS compared to treatment with sorafenib, which is recommended as the first-line treatment in westernized countries. HAIC can transport anticancer agents to tumors at high local concentrations and reduce dissemination to areas unaffected by cancer. In addition, many studies' findings [15–18] have demonstrated that HAIC effectively treats patients with HCC and PVTT and that it significantly prolongs a patient's survival.

The administration of HAIC and TACE in combination [19–22] has been proposed based on its effectiveness at treating advanced HCC and its safety and ability to prolong OS and PFS among patients with inoperable and advanced HCC. TACE almost completely occludes the vessels supplying tumors, which reduces the dilution of a perfused chemotherapeutic drug and prolongs the duration of action of the anticancer agent [22].

In addition, a study' finding [23] has shown that among patients with HCC and major PVTT, treatment with TACE and HAIC was effective, and it did not increase major complication rates. However, the outcomes of combined therapy comprising conventional TACE (cTACE) and HAIC and cTACE administered alone have not been compared in patients with HCC and PVTT. This study was aimed at comparing the treatment outcomes of cTACE and HAIC administered in combination and cTACE administered alone among patients with HCC and PVTT.

## 2. Materials and Methods

### 2.1. Study Design and Patient Selection

This single-center, retrospective study was approved by our hospital's research ethics committee, and the requirement for informed consent was waived because of the retrospective design of this study. The study was conducted in accordance with the principles of the Declaration of Helsinki.

**Figure 1:**
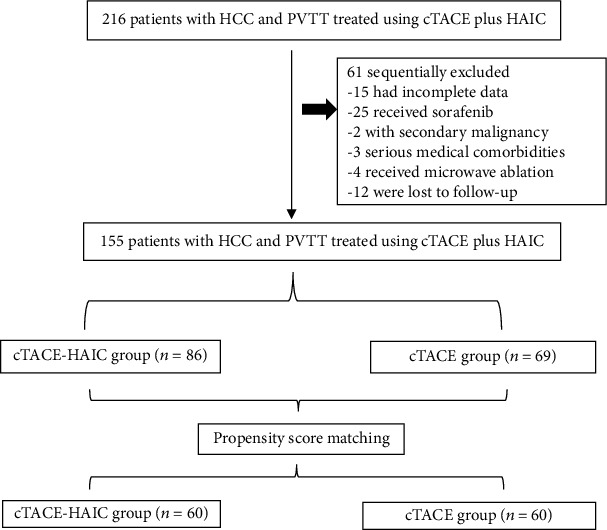
Flow diagram showing patient selection for the study. HCC: hepatocellular carcinoma; cTACE: conventional transarterial chemoembolization; HAIC: hepatic arterial infusion chemotherapy; PVTT: portal vein tumor thrombosis.

We reviewed data from 216 patients with HCC and PVTT who underwent cTACE alone (cTACE group) or cTACE and HAIC in combination (cTACE-HAIC group) from January 2011 to December 2016. The final follow-up assessment was conducted on December 30, 2019. Of these patients, 61 were excluded, because 25 had received sorafenib; 15 had incomplete data; 2 had additional tumors; 3 had serious medical comorbidities, including pulmonary embolism, right atrial thrombus, and increased bilirubin levels; four had undergone microwave ablation during artery-directed therapy; and 12 were lost to follow-up. Consequently, a total of 155 patients were included in the final analysis, of whom, 86 patients received cTACE and HAIC in combination and 69 patients received cTACE alone (Figure 1). All patients were diagnosed based on their pathology findings or using the criteria of the American Association for the Study of Liver Disease (AASLD) [24]. The extent of portal vein invasion was classified into four types, namely, VP1–VP4, according to the Liver Cancer Study Group of Japan's criteria [25]. Patients were included if they were aged 18–85 years and had an adequate bone marrow count, which was defined as a white blood cell count > 3.0 × 10^9^/L or an absolute neutrophil count >1.5 × 10^9^/L, a platelet count of 60 × 10^9^/L, hepatic alanine aminotransferase (ALT) and aspartate aminotransferase (AST) levels ≤ 5 times the upper limit of normal, a serum creatinine level ≤ 2.0 mg/dL and a renal creatinine clearance ≤ 1.5 times the upper limit of the normal, an international normalized ratio ≤ 1.5, a Child–Pugh grade A or B, at least one measurable intrahepatic lesion according to the modified response evaluation criteria in solid tumors (mRECIST) [26], and an Eastern Cooperative Oncology Group − Performance Status (ECOG − PS) ≤ 2; adequate collateral circulation from the anterior circulation must be indicated, when the portal tumor thrombus completely filled the major portal vein. On the other hand, the exclusion criteria were as follows: (1) patients with prior or concomitant malignancies, (2) those with diffuse lesions of HCC and with upper gastrointestinal bleeding history or ascites, (3) a left ventricular ejection fraction ≤ 45%, (4) patients with missing data on their first imaging assessments, and (5) those who were lost to follow-up.

### 2.2. Conventional Transarterial Chemoembolization

The Seldinger technique was used to access the femoral artery after an injection of a local anesthetic. Digital subtraction angiography (DSA) (Innova 4100IQ; General Electric Company, Boston, MA, USA) was performed to visualize the celiac, superior mesenteric, and splenic arteries and the hepatic arterial anatomy and to evaluate the tumor's blood supply. The portovenous flow was evaluated during the portal venous phases of the superior mesenteric or splenic angiograms. After detecting the tumors' vessels and the arteries supplying the tumors, a 2.7 F microcatheter was inserted coaxially into the tumor feeding arteries for superselective embolization involving an emulsion of lipiodol (5–15 mL) (Lipiodol® Ultra Fluid; Guerbet, Aulnay-Sous-Bois, France) and epirubicin (40–60 mg) under fluoroscopic guidance. Preserving the blood flow of the main artery to perform HAIC and avoid increased portal hypertension, the cTACE is incomplete embolism, and the tumors were subsequently infused with chemotherapeutic agents. It should be noted that established collateral circulation and good liver function preservation is important for cTACE. For some large tumors, the embolization was done two or three times to prevent the hepatic infarction or failure. Collateral artery embolization was performed if branches of the phrenic artery and internal thoracic artery comprised a tumor's blood supply. Embolization using 350–1000 *μ*m gelatin sponge particles was performed in patients with arterioportal shunts (to occlude the shunts via superselective catheterization) before the lipiodol infusion.

### 2.3. Conventional Transarterial Chemoembolization and Hepatic Arterial Infusion Chemotherapy

For the patients who underwent cTACE and HAIC, cTACE was performed, as previously described. Subsequently, the catheter was retained in the segmental artery, and its placement was confirmed appropriate using DSA. Following the patient's return to the ward, the microcatheter was externally connected to an artery infusion pump to administer the HAIC that comprised oxaliplatin (OXA) (85 mg/m^2^) administered intra-arterially for 4 h, leucovorin (200 mg/m^2^) administered intravenously for 2 h, and 5-fluorouracil (5-FU) (1.5 g/m^2^) administered intra-arterially for 20 h. Treatment was repeated every 6-8 weeks, and it continued until the intrahepatic lesions progressed or toxicity became unacceptable. The procedure of cTACE plus HAIC in the study is similar to cTACE-HAIC in a previous study [21, 27].

### 2.4. Follow-Up

The criteria for discontinuing treatment included radiographic or symptomatic progression; unacceptable toxicity; disease downstaging that facilitated surgery, ablation, or liver transplantation; death; and a patient's refusal to continue. Safety was assessed among all the patients treated. Before each treatment cycle and within 1 week after treatment, the patients underwent physical examinations and blood tests to assess their hematologic profiles, renal function, and liver function and to perform coagulation screens. In addition, every two weeks after treatment, the patients underwent routine tests that included blood tests to assess their hematologic profiles, renal function, and liver function. The patients were evaluated using abdominal contrast-enhanced three-phase dynamic spiral computed tomography (CT) or magnetic resonance imaging (MRI) and chest CT and bone scanning within 1 week before treatment, if applicable, after every 1 cycle of treatment. We tried to maintain consistency in imaging examination (CT or MRI) before and after treatment.

### 2.5. Tumor Response and Survival Assessments

The study's primary endpoint was overall survival (OS), which was defined as the interval between the time of treatment initiation and death or the last follow-up assessment. The secondary endpoints were progression-free survival (PFS), tumor response rates, and safety. PFS was defined as the interval between the time of treatment initiation and intra- or extrahepatic tumor progression; symptomatic progression, including massive ascites and liver function that was categorized as Child–Pugh grade C; or death from any cause. A tumor's response was assessed using the mRECIST [26]. The objective response rate (ORR) was defined as the complete response (CR) rate + the partial response (PR) rate, and the disease control rate (DCR) was defined as the CR rate + the PR rate + the stable disease (SD) rate. Based on a clinical study, progressive disease was categorized as intrahepatic progression or extrahepatic progression [17]. Two experienced radiologists with 13 and 14 years of experience in abdominal imaging determined the tumors' responses by consensus.

The times of cTACE-HAIC vary according to the responses of treatment and characterization of tumors. Evaluation of the effect of therapy was performed every 4 to 6 weeks by enhanced MRI or CT. If the effect was CR, the treatment was stopped and the subsequent evaluation will be performed in the next 4 to 6 weeks to confirm the response. If the response were PR or SD, the treatment will be performed for 4 to 6 cycles according to liver function and collateral circulation around the occluded portal veins, and the evaluation will be performed subsequently every 4 to 6 weeks. However, if the tumor progressed after 4 to 6 treatment cycles, the treatment will be restarted. If the response was PD after the first treatment, cTACE-HAIC will be stopped, and a new treatment will be performed.

### 2.6. Statistical Analyses

The Wilcoxon rank-sum test and the independent *t*-test were used to analyze the continuous variables, whereas the chi-square test and Fisher's exact test were used to analyze the categorical variables. Propensity score matching (PSM) [28] was used to reduce selection bias and the effects of potential confounders associated with the clinical characteristics of the groups. The propensity scores were estimated using logistic regression that predicted the probability of patients being classified under the cTACE-HAIC group. Standardized mean differences <0.10 indicated minimal differences and achieved a balance in the variables, including age, sex, and the disease etiology, type of portal vein invasion, Child–Pugh score, ECOG-PS score, intrahepatic tumor size, extrahepatic spread, and the alpha-fetoprotein level.

OS and PFS were estimated using the Kaplan–Meier method with the log-rank test and Cox proportional hazard regression models. The statistical analyses were conducted using IBM®SPSS® software, version 22.0 (IBM Corporation, Armonk, NY, USA) and R, version 2.15.x (The R Foundation for Statistical Computing, Vienna, Austria). Differences with values of *p* < 0.05 were considered statistically significant.

## 3. Results

### 3.1. Patients' Characteristics

Table 1 presents the baseline characteristics of all patients with HCC (*n* = 155) involved in this study. The mean age of patients was 54.4 ± 10.9 years (range: 27–86 years). In addition, 133 (85.8%) patients were infected with the hepatitis B virus, nine (5.81%) patients were infected with the hepatitis C virus, and 143 (92.2%) patients were categorized as Child–Pugh class A. Further, 12 (7.7%) patients were categorized as Child–Pugh class B, 16 (10.3%) patients had their PVTT classified as VP1–VP2, 139 (89.7%) had their PVTT classified as VP3–VP4, and 58 (37.4%) patients presented with extrahepatic spread. Ablation (*n* = 5), cTACE (*n* = 4), radiotherapy (*n* = 1), and implantation therapy (*n* = 1) had been performed for patients in both treatment groups before study enrollment. The cTACE group had a higher rate of VP1–VP2 PVTT (17.4%) than the cTACE-HAIC group (4.7%) (*p* = 0.019). One-to-one PSM produced 60 matched patient pairs.  Table 2 summarizes the demographic characteristics of the patients after PSM (*n* = 120); the two groups were well balanced regarding the baseline characteristics. The median follow-up durations were 93 months and 57 months for the cTACE-HAIC and cTACE groups, respectively.

### 3.2. Overall Survival

**Figure 2:**
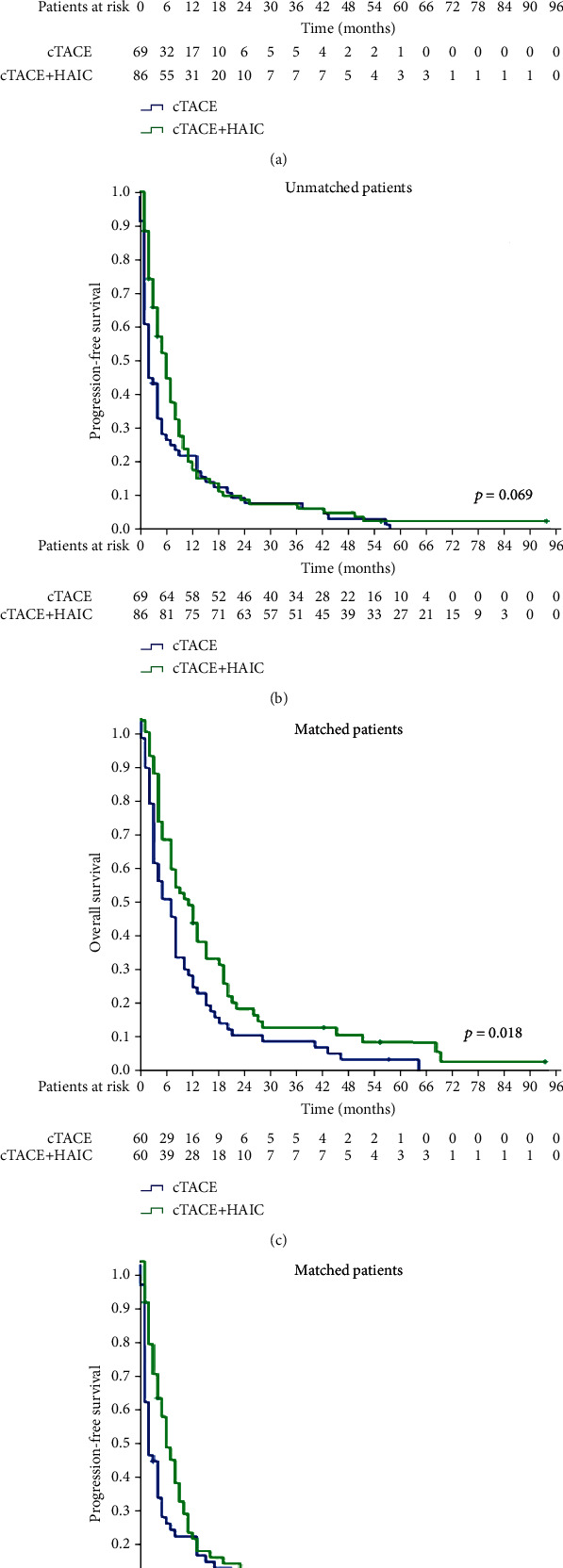
Kaplan-Meier curves showing overall survival (OS) and progression-free survival (PFS) in the unmatched groups of patients who received conventional transarterial chemoembolization (cTACE) and hepatic arterial infusion chemotherapy (HAIC) in combination or cTACE only. (a) OS in the unmatched cohort. OS was significantly longer for the patients who were treated with cTACE and HAIC than that for the patients who received cTACE only in the unmatched cohort (*p* = 0.045). (b) PFS in the unmatched cohort. PFS was longer for the patients who received cTACE and HAIC compared with that for the patients who received cTACE alone (*p* = 0.069). (c) OS in the matched cohort. OS was significantly longer for the patients who received cTACE and HAIC than for the patients who received cTACE alone in the matched cohort (*p* = 0.018). (d) PFS in the matched cohort. PFS was significantly longer for the patients who received cTACE and HAIC than for the patients who received cTACE alone in the matched cohort (*p* = 0.045). TACE: transcatheter arterial chemoembolization; HAIC: hepatic arterial infusion chemotherapy.

The median follow-up duration was 93 months (range: 1–93 months) for all patients. During follow-up, 151 of 155 patients died, comprising 82 of 86 (95.3%) patients in the cTACE-HAIC group and 68 of 69 (98.6%) patients in the cTACE group. The leading cause of death was cancer progression (*n* = 145); similarly, death was also caused by renal failure (*n* = 1), gastrointestinal bleeding (*n* = 3), cerebrovascular disease (*n* = 1), and unknown causes (*n* = 1). The median OS in the cTACE-HAIC group (8 months) was longer than that in the cTACE group (5 months) before PSM (*p* = 0.043) (Figure 2(a)). After PSM, the median OS significantly differed between the cTACE-HAIC group (9 months) and the cTACE group (5 months) (*p* = 0.018) (Figure 2(c)). In the subgroup analysis of patients with VP3-VP4 after PSM, the median OS was significantly longer in the cTACE-HAIC group (10 months) and the cTACE group (4 months) (*p* = 0.004). Multivariate analyses of the matched cohort (*n* = 120) showed that cTACE-HAIC (hazard ratio (HR) 0.60, 95% confidence interval (CI) 0.409–0.887, *p* = 0.010) and the albumin (ALB) grade (HR 3.11, 95% CI 1.488–6.502, *p* = 0.003) were independent prognostic factors associated with OS (Table 3).

### 3.3. Progression-Free Survival

Overall, 147 patients experienced disease progression that comprised intrahepatic (*n* = 133), lung (*n* = 4), lung and intrahepatic (*n* = 3), lymph node (*n* = 2), and symptomatic (*n* = 5) progressions. As the disease progressed, different treatments were implemented, including radiotherapy (*n* = 10), sorafenib (*n* = 5), particle implantation (*n* = 1), and systemic chemotherapy (*n* = 1). Before PSM, the median PFS of the cTACE-HAIC group (6 months) was longer than that of the cTACE group (2 months) (*p* = 0.069) (Figure 2(b)). After PSM, the median PFS of the cTACE-HAIC group (6 months) was significantly longer than that of the cTACE group (2 months) (*p* = 0.045) (Figure 2(d)). After PSM, the median PFS of patients with VP3-VP4 is differed between the cTACE-HAIC group (6 months) and the cTACE group (2 months) (*p* = 0.010). Multivariate analyses of the propensity score-matched cohort (*n* = 120) showed that cTACE and HAIC (HR 0.67, 95% CI 0.453–0.977, *p* = 0.038) and the ALB grade (HR 2.12, 95% CI 1.018–4.407, *p* = 0.045) were independent prognostic factors associated with PFS (Table 3).

### 3.4. Tumor Responses

**Figure 3:**
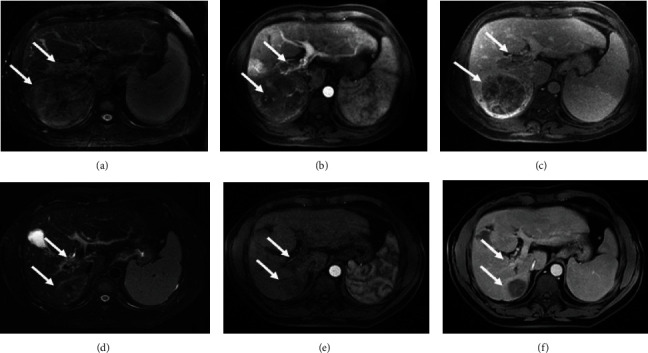
Images from a 45-year-old man with advanced hepatocellular carcinoma (HCC) with Vp3 portal vein tumor thrombosis (PVTT). (a–c) The right main portal vein thrombosis and a huge lesion (84 × 81 mm) in the right lobe of the liver were revealed by MRI T2 phase and contrast-enhanced MRI (CE-MRI) before chemoembolization and hepatic arterial infusion chemotherapy (TACE-HAIC) (arrows). (b–d) After six cycles of TACE-HAIC, MRI T2 phase and CE-MRI imaging showed that the tumor thrombosis and liver lesion were further reduced in size (arrows) with almost no enhancement. The overall survival (OS) was 93.1 months.

Table 4 shows the tumor responses in patients with HCC and PVTT. Based on the mRECIST, the ORR (CR+PR) of the cTACE-HAIC group was significantly higher than that of the cTACE group in both the unmatched (*p* = 0.002) and matched (*p* < 0.001) cohorts; additionally, the DCR (CR+PR+SD) of the cTACE-HAIC group was significantly higher than that of the cTACE group in both the unmatched (*p* = 0.001) and matched (*p* = 0.002) cohorts. A patient who underwent the TACE-HAIC is shown in Figure 3.

### 3.5. Safety

The mean treatment duration of patients was 2.4 cycles (range: 1–9 cycles). No difference was observed between groups regarding the frequency of grade 3 or 4 adverse effects after PSM (Table 5). Grade 3 or 4 adverse effects were observed in 14 of the 60 (23.3%) patients in the cTACE-HAIC group and in 13 of the 60 (21.7%) patients in the cTACE group (*p* = 0.827). Although no difference was observed in the grade 3 or 4 adverse event rate between groups, the percentage of patients who experienced grade 1 or 2 adverse events was higher in the cTACE-HAIC group than in the cTACE group, which included vomiting (41.7% vs. 21.7%, *p* = 0.019) and thrombocytopenia (43.3% vs. 28.3%, *p* = 0.087). Treatment with cTACE or cTACE and HAIC was not interrupted as a consequence of grade 3 or 4 adverse events. The adverse events were generally manageable in the propensity score-matched groups. The most common grade 1 or 2 adverse events in the cTACE-HAIC group of the propensity score-matched cohort included fever in 68.3% of the patients and liver dysfunction that was indicated by elevated levels of ALT in 60.0%, AST in 73.3%, and total bilirubin in 76.7% of the patients. Severe vascular complications were not observed. No treatment-related deaths were observed in either group within 2 weeks from treatment initiation.

## 4. Discussion

The study findings revealed that compared with the treatment comprising cTACE alone, cTACE plus HAIC administered in combination was associated with significant survival benefits and improved tumor responses among patients with HCC and PVTT; additionally, the adverse events caused by cTACE plus HAIC were tolerable and expected. The credibility of these findings was substantiated by PSM that mimics randomization, which may be undertaken in prospective studies and reduces bias caused by confounding variables [29].

According to the European Association for the Study of the Liver (EASL) guidelines, macrovascular invasion of the main portal vein is a contraindication for TACE. Sorafenib is the standard therapy for advanced HCC in westernized countries. But sorafenib showed an OS of only 6.5 months for patients with advanced HCC in Asia [30], and the Japan Society of Hepatology (JSH) guidelines state that is not considered a contraindication for TACE. Sorafenib was not included in the Chinese health insurance policies between 2011 and 2016; it is cost-ineffective for patients and their families, and many patients have not used sorafenib in China. Therefore, cTACE is an alternative treatment for advanced HCC in China. HAIC is an effective treatment for HCC with PVTT. cTACE plus HAIC may benefit patients with HCC and PVTT. Comparing with cTACE alone, cTACE plus HAIC were observed to demonstrate significantly longer OS and PFS for HCC with vascular invasions.

In some randomized controlled trials (RCTs), the safety and effectiveness of cTACE has been confirmed in patients with HCC invading the main portal vein [7]. Our previous work indicated that cTACE-HAIC is a safe and effective treatment for advanced HCC [21, 23, 27]. The mechanism underlying the combination treatment with cTACE and HAIC is subsequently described. First, cTACE delays the flow within the artery supplying the tumor, especially when a tumor has an arterioportal shunt; this exposes the tumor to high concentrations of drugs for longer durations. Second, cTACE induces tumor tissue ischemia and hypoxia and tumor cell transmembrane ion pump failure that reduces the discharge of chemotherapeutic agents from the tumor tissue [31]. For drug-resistant tumors, cTACE with HAIC may improve treatment efficacy. Finally, HAIC can clear most of the residual lesions that persist after TACE.

However, it is necessary to carefully select patients who can be treated. The established collateral circulation and good liver function preservation (Child–Pugh A-B) are more important than the location of PVTT (VP1-VP4). Adequate collateral circulation around the occluded portal veins can decrease the risk of ischemia-induced hepatic insufficiency. TACE is contraindicated for patients with severe portal hypertension (upper gastrointestinal bleeding and ascites due to portal hypertension). Second, the embolization strategy is important for HCC with PVTT. Incomplete embolization is also very important for HCC with PVTT; for some large tumors or multiple tumors, the embolization was done for two or more times to prevent hepatic infarction or hepatic failure. For diffuse lesions of HCC, TACE will be done carefully or will be abandoned to prevent hepatic failure; they are excluded from this study. In addition, we incompletely embolized lesions in the liver and PVTTs through the hepatic artery branches to decrease the risk of hepatic failure. Third, extra care should be taken in choosing the embolic material. For example, large diameter particles should be avoided for these patients.

In our study, cTACE-HAIC was repeated in 4 to 6 weeks for HCC with PVTT and was accepted by 95% of patients. The interval of 4 to 6 weeks provides ample time for the restoration of liver function. It also gives sufficient time for patients to rest from adverse effects. From the perspective of clinical economics, the longer cycle of therapy at 4 to 6 weeks is more cost-effective.

In this study, the median OS (9.0 months) after treatment with cTACE and HAIC was equally higher than the findings from a prospective comparative study of TACE (7.1 months) [7] and similar to the OS reported by previous studies of TACE+HAIC [22, 23]. The findings from a study analyzing patients with HCC and PVTT who underwent HAIC showed that the median OS (7.1 months) and PFS (3.3 months) were shorter than those obtained in this study [32]. A previous study [18] of our center investigated the effects of HAIC versus TACE/TAE administered to HCC with major PVTT and showed that the median OS in the HAIC group and TACE/TAE group were 20.8 months and 4.0 months, respectively. The OS of HAIC alone was longer than this study. The possible reasons were that patients in the previous study had more frequent chemotherapy (every 4 weeks) and less tumor load (mean tumor size); in addition, the small sample size may introduce more bias.

The overall toxicity of the treatments administered to the patients in this study was acceptable. Leucovorin, 5-FU, and OXA (FOLFOX) are safe and effective chemotherapy regimens for advanced HCC [33]. Moreover, some studies have shown HAIC with FOLFOX is a safe and effective treatment for advanced HCC [17, 34]. OXA is a new generation of platinum-based chemotherapy drugs, and it is safer and more effective than cisplatin for HCC [35, 36] .The incidence of grade 1 or 2 vomiting was significantly higher in the cTACE-HAIC group than in the cTACE group (*p* = 0.019), which may have been associated with the administration of OXA and 5-FU. The grade 3 or 4 adverse event rate was similar to the rates reported from previous studies that implemented HAIC or TACE for advanced HCC [14, 17, 37]. In addition, compared with the rate reported from the EACH study (55.7%) [33], the grade 3 or 4 adverse event rate in this study was significantly low. These adverse events were expected and could be managed by dosage adjustments.

### 4.1. Limits of the Study

This study has several limitations. First, this was a retrospective study that was conducted at a single center and involved a limited sample size; therefore, its findings were subject to bias, which must be considered during interpretation. Although the selection bias was reduced using PSM, the effects of unmeasured confounding variables cannot be excluded. Second, to verify the accuracy of the study data, many patients were excluded from the sample, and reducing the sample size may have introduced bias. Third, although PSM and multivariate analyses were performed to balance the groups in relation to these differences, both groups could have been affected by unidentified biases. Finally, while the operators were experienced in administering cTACE and HAIC, differences regarding the physicians' experience might have exacerbated the bias. Therefore, large prospective multicenter and randomized controlled trials are required to confirm the results of this study.

## 5. Conclusion

In conclusion, these study findings indicated that for patients with HCC with PVTT, cTACE and HAIC administered in combination was superior to cTACE administered alone with regard to PFS and OS. The treatment toxicities were generally well tolerated.

## Figures and Tables

**Table 1:** Patient baseline demographic and clinical characteristics (unmatched).

Characteristics	cTACE (*n* = 69)	cTACE+HAIC (*n* = 86)	*p* value	Standardized mean difference
Age (years)^†^	54.07 ± 11.54	54.80 ± 10.43	0.680	0.058
Sex			0.963	0.008
Male	60 (87.0)	75 (87.2)		
Female	9 (13.0)	11 (12.8)		
Etiology			0.883	0.020
Hepatitis B virus	60 (87.0)	73 (84.9)		
Hepatitis C virus	3 (4.3)	6 (7.0)		
Unknown/other	6 (8.7)	7 (8.1)		
Cirrhosis clinical course			0.496	
Stage 1-2	59 (85.5)	70 (81.4)		
Stage 3-4	10 (14.5)	16 (18.6)		
Child-Pugh class			0.316	0.184
A	62 (89.9)	81 (94.2)		
B	7 (10.1)	5 (5.8)		
ALB grade			0.417	
0	65 (94.2)	78 (90.7)		
1-2	4 (5.8)	8 (9.3)		
ECOG performance status			0.336	0.153
0	43 (62.3)	47 (54.7)		
1-2	26 (37.7)	39 (45.3)		
Portal vein invasion stage			0.019	0.173
Vp1-2	12 (17.4)	4 (4.7)		
Vp3	30 (43.5)	51 (59.3)		
Vp4	27 (39.1)	31 (36.0)		
Extrahepatic spread			0.952	0.010
Absent	43 (62.3)	54 (62.8)		
Present	26 (37.7)	32 (37.2)		
Tumor diameter (mm)			0.520	0.104
>100	30 (43.5)	34 (39.5)		
≤100	39 (56.5)	52 (60.5)		
Mean ± SD (mm)^†^	99.28 ± 46.79	95.22 ± 46.32	0.622	
AFP (ng/mL)^‡^	2429.50 (17.74 − 7.8 × 10^5^)	3919.00 (12.41 − 1.9 × 10^6^)	0.454	0.066
≤1000	29 (42.0)	39 (45.3)	0.679	
>1000	40 (58.0)	47 (54.7)		

ALB: albumin; TACE: transcatheter arterial chemoembolization; HAIC: hepatic arterial infusion chemotherapy; AFP: serum *α*-fetoprotein level; SD: standard deviation. ^†^Data mean ± standard deviation. ^‡^Data were median (full range). Unless indicated otherwise, data are the number of patients, with percentages in parentheses. Continuous variables were analyzed using the two-sample *t* test, or the Wilcoxon rank-sum test was used. Categorical variables were compared by using the *χ*^2^ test.

**Table 2:** Patient baseline demographic and clinical characteristics (matched).

Characteristics	cTACE (*n* = 60)	cTACE+HAIC (*n* = 60)	*p* value	Standardized mean difference^∗^
Age (years)^†^	53.63 ± 11.47	54.67 ± 9.47	0.510	0.036
Sex			0.789	0.099
Male	51 (85.0)	53 (88.3)		
Female	9 (15.0)	7 (11.7)		
Etiology			0.714	0.061
Hepatitis B virus	54 (90.0)	53 (88.3)		
Hepatitis C virus	3 (5.0)	3 (5.0)		
Unknown/other	3 (5.0)	4 (6.7)		
Cirrhosis clinical course				
Stage 1-2	54 (90.0)	49 (81.7)	0.295	
Stage 3-4	6 (10.0)	11 (18.3)		
Child-Pugh class			1.000	0.000
A	56 (93.3)	56 (93.3)		
B	4 (6.7)	4 (6.7)		
ALB grade			0.163	
0	58 (96.7)	53 (88.3)		
1-2	2 (3.3)	7 (11.7)		
ECOG performance status			1.000	0.000
0	36 (60.0)	36 (60.0)		
1-2	24 (40.0)	24 (40.0)		
Portal vein invasion stage			0.138	0.000
Vp1-2	8 (13.3)	3 (5.0)		
Vp3	28 (46.7)	38 (63.3)		
Vp4	24 (40.0)	19 (31.7)		
Extrahepatic spread			0.709	0.069
Absent	35 (58.3)	37 (61.7)		
Present	25 (41.7)	23 (38.3)		
Tumor diameter (mm)			0.852	0.068
>100	25 (41.7)	23 (38.3)		
≤100	35 (58.3)	37 (61.7)		
Mean ± SD (mm)^†^	96.98 ± 45.21	93.83 ± 45.49	0.747	
AFP (ng/mL)^‡^	3000 (17.74 − 7.8 × 10^5^)	4808 (20 − 7.4 × 10^5^)	0.282	0.000
≤1000	26 (43.3)	26 (43.3)	1.000	
>1000	34 (56.6)	34 (56.6)		

ALB: albumin; TACE: transcatheter arterial chemoembolization; HAIC: hepatic arterial infusion chemotherapy; AFP: serum *α*-fetoprotein level; SD: standard deviation. ^†^Data mean ± standard deviation. ^‡^Data were median (full range). Unless indicated otherwise, data are the number of patients, with percentages in parentheses. Continuous variables were analyzed using the two-sample *t* test, or the Wilcoxon rank-sum test was used. Categorical variables were compared by using the *χ*^2^ test.

**Table 3:** Prognostic factor analysis for progression-free survival and overall survival (matched).

Variable	Overall survival				Progression-free survival			
	Univariate analysis		Multivariate analysis		Univariate analysis		Multivariate analysis	
	HR (95% CI)	*p*	HR (95% CI)	*p*	HR (95% CI)	*p*	HR (95% CI)	*p*
Treatment type	0.65 (0.451-0.947)	0.018	0.60 (0.409-0.887)	0.010	0.70 (0.485-1.018)	0.045	0.67 (0.453-0.977)	0.038
Age (years)	1.05 (0.700-1.587)	0.801			0.94 (0.621-1.435)	0.788		
Cirrhosis clinical course	1.04 (0.619-1.748)	0.882			1.08 (0.640-1.810)	0.782		
Child-Pugh class	2.41 (1.162-5.001)	0.018	1.97 (0.941-4.138)	0.072	2.33 (1.123-4.840)	0.023	1.93 (0.913-4.067)	0.085
ALB grade	0.95 (0.913-0.991)	0.017	3.11 (1.488-6.502)	0.003	0.96 (0.915-0.996)	0.032	2.12 (1.018-4.407)	0.045
ECOG PS	0.87 (0.595-1.260)	0.453			0.90 (0.616-1.315)	0.586		
Portal vein invasion stage	1.10 (0.819-1.482)	0.523			1.08 (0.807-1.449)	0.600		
Extrahepatic spread	1.20 (0.826-1.748)	0.337			1.16 (0.797-1.686)	0.439		
Tumor diameter	0.967 (0.667-1.401)	0.859			0.85 (0.586-1.238)	0.400		
AFP	1.14 (0.788-1.656)	0.482			1.05 (0.721-1.517)	0.813		

ALB: albumin; HR: hazard ratio; OS: overall survival; PFS: progression-free survival; AFP: serum *α*-fetoprotein level. Data in parentheses are 95% confidence intervals. A Cox proportional hazard regression model for PFS and OS was used.

**Table 4:** Comparison of efficacy of TACE-HAIC with TACE group based on tumor response.

Response (by mRECIST)	Pooled cohort (*n* = 155)			Matched cohort (*n* = 120)		
	cTACE (*n* = 69)	cTACE+HAIC (*n* = 86)	*p* value	cTACE (*n* = 60)	cTACE+HAIC (*n* = 60)	*p* Value
Complete	0 (0.0)	3 (3.5)	<0.001	0 (0.0)	3 (5.0)	<0.001
Partial	4 (5.8)	18 (20.9)	0.001	2 (3.3)	16 (26.7)	<0.001
Stable disease	33 (47.8)	47 (54.7)	0.007	31 (51.7)	30 (50.0)	0.855
Progressive disease	32 (46.4)	18 (20.9)	0.010	27 (45.0)	11 (18.3)	<0.001
Objective response rate	4 (5.8)	21 (24.4)	0.002	2 (3.3)	19 (31.7)	<0.001
Disease control rate	37 (53.6)	68 (79.1)	0.001	33 (55.0)	49 (81.7)	0.002

TACE: transcatheter arterial chemoembolization; HAIC: hepatic arterial infusion chemotherapy; mRECIST: modified response evaluation criteria in solid tumors. Objective response rate = complete response + partial response. Disease control rate = complete response + partial response + stable disease. Statistical significance was assessed with the *χ*^2^ test or Fisher's exact test.

**Table 5:** Treatment-related adverse events in the groups (matched).

	cTACE group (*n* = 60)	cTACE+HAIC group (*n* = 60)	*p* value
Any grade 3-4	13 (21.7)	14 (23.3)	0.827
*Blood suppression*			
Leukopenia			
Grades 1 to 2	6 (10.0)	6 (10.0)	1.000
Grades 3 to 4	0 (0.0)	0 (0.0)	1.000
Neutropenia			
Grades 1 to 2	2 (3.3)	3 (5.0)	0.5
Grades 3 to 4	0 (0.0)	0 (0.0)	1.000
Reduced hemoglobin			
Grades 1 to 2	12 (20.0)	14 (23.3)	0.658
Grades 3 to 4	1 (1.6)	2 (3.3)	0.500
Thrombocytopenia			
Grades 1 to 2	17 (28.3)	26 (43.3)	0.087
Grades 3 to 4	2 (3.3)	2 (3.3)	1.000
*Constitutional symptom*			
Fever			
Grades 1 to 2	43 (71.7)	41 (68.3)	0.690
Grades 3 to 4	3 (5.0)	0 (0.0)	0.244
*Indigestion*			
Vomiting			
Grades 1 to 2	13 (21.7)	25 (41.7)	0.019
Grades 3 to 4	0 (0)	0 (0.0)	1.000
*Hepatic function*			
Elevated ALT			
Grades 1 to 2	49 (81.7)	36 (60.0)	0.009
Grades 3 to 4	5 (8.3)	7 (11.7)	0.543
Elevated AST			
Grades 1 to 2	50 (83.3)	44 (73.3)	0.184
Grades 3 to 4	6 (10.0)	7 (11.7)	0.769
Elevated TBIL			
Grades 1 to 2	41 (68.3)	46 (76.7)	0.307
Grades 3 to 4	9 (15.0)	3 (5.0)	0.068
*Hypertension*			
Grades 1 to 2	9 (15.0)	12 (20.0)	0.471
Grades 3 to 4	0 (0.0)	0 (0.0)	1.000
*Pain*			
Grades 1 to 2	40 (66.7)	42 (70.0)	0.695
Grades 3 to 4	0 (0)	0 (0.0)	1.000

TACE: transcatheter arterial chemoembolization; HAIC: hepatic arterial infusion chemotherapy; ALT: alanine aminotransferase; AST: aspartate aminotransferase; TBIL: total bilirubin. Statistical significance was assessed with the *χ*^2^ test or Fisher's exact test.

## Data Availability

The data used to support the findings of this study are available from the corresponding author upon request.
